# Deficiency of Ataxia Telangiectasia Mutated Kinase Modulates Cardiac Remodeling Following Myocardial Infarction: Involvement in Fibrosis and Apoptosis

**DOI:** 10.1371/journal.pone.0083513

**Published:** 2013-12-16

**Authors:** Cerrone R. Foster, Laura L. Daniel, Christopher R. Daniels, Suman Dalal, Mahipal Singh, Krishna Singh

**Affiliations:** Department of Biomedical Sciences, James H Quillen College of Medicine, James H Quillen Veterans Affairs Medical Center, East Tennessee State University, Johnson City, Tennessee, United States of America; INSERM, France

## Abstract

Ataxia telangiectasia mutated kinase (ATM) is a cell cycle checkpoint protein activated in response to DNA damage. We recently reported that ATM plays a protective role in myocardial remodeling following β-adrenergic receptor stimulation. Here we investigated the role of ATM in cardiac remodeling using myocardial infarction (MI) as a model. **Methods and Results:** Left ventricular (LV) structure, function, apoptosis, fibrosis, and protein levels of apoptosis- and fibrosis-related proteins were examined in wild-type (WT) and ATM heterozygous knockout (hKO) mice 7 days post-MI. Infarct sizes were similar in both MI groups. However, infarct thickness was higher in hKO-MI group. Two dimensional M-mode echocardiography revealed decreased percent fractional shortening (%FS) and ejection fraction (EF) in both MI groups when compared to their respective sham groups. However, the decrease in %FS and EF was significantly greater in WT-MI vs hKO-MI. LV end systolic and diastolic diameters were greater in WT-MI vs hKO-MI. Fibrosis, apoptosis, and α-smooth muscle actin staining was significantly higher in hKO-MI vs WT-MI. MMP-2 protein levels and activity were increased to a similar extent in the infarct regions of both groups. MMP-9 protein levels were increased in the non-infarct region of WT-MI vs WT-sham. MMP-9 protein levels and activity were significantly lower in the infarct region of WT vs hKO. TIMP-2 protein levels similarly increased in both MI groups, whereas TIMP-4 protein levels were significantly lower in the infarct region of hKO group. Phosphorylation of p53 protein was higher, while protein levels of manganese superoxide dismutase were significantly lower in the infarct region of hKO vs WT. In vitro, inhibition of ATM using KU-55933 increased oxidative stress and apoptosis in cardiac myocytes.

## Introduction

Myocardial infarction (MI) induces a series of molecular and structural changes in the left ventricle leading to a progressive decline in LV performance [1–3]. The limited capacity for regeneration of myocytes in the adult heart suggests that cardiac myocyte loss due to apoptosis may contribute to the progression of heart failure. Dynamic synthesis and breakdown of extracellular matrix also plays a significant role in myocardial remodeling post-MI [4,5]. Therefore elucidation of events involved in the repair of the heart is an important clinical determinant of survival post-MI [6]. 

Ataxia telangiectasia mutated kinase (ATM) is a multifunctional kinase that affects multiple downstream targets in response to cellular stress or damage. Mutation or deficiency of ATM causes a hereditary multi-systemic disease called Ataxia telangiectasia (A-T). Individuals with mutations in both copies of the ATM gene suffer from increased susceptibility to ionizing radiation, predisposition to cancer, insulin resistance, immune deficiency, and premature aging. Carriers of one mutated allele at the A-T locus make up ~1.4 to 2% of the general population. These individuals with an ATM mutation in one allele are spared from most of the symptoms of A-T, but are more susceptible to cancer and ischemic heart disease [7–9]. 

Previously, a search to identify novel apoptosis-related genes using Super-Array technique followed by RT-PCR analyses revealed that β-adrenergic receptor (β-AR) stimulation increases expression of ATM in the heart and in adult cardiac myocytes [10]. Using ATM heterozygous knockout (hKO) mice and chronic β-AR stimulation as a model of myocardial remodeling, we provided evidence that ATM plays an important role in β-AR-stimulated myocardial remodeling with effects on ventricular function, apoptosis and fibrosis [10]. Recently, using ATM-/- mice, we have shown that lack of ATM induces structural and functional changes in the heart with enhanced myocardial fibrosis and myocyte hypertrophy. β-AR-stimulated apoptosis in WT hearts associated with p53- and JNKs-dependent mechanism, while decreased Akt activity may play a role in increased myocyte apoptosis in the absence of ATM [11]. The objective of this study was to investigate the role of ATM in myocardial remodeling 7 days post-MI. The data presented here show that deficiency of ATM affects heart function, infarct thickness, fibrosis, apoptosis and expression of fibrosis- and apoptosis-related proteins. 

## Methods

### Vertebrate animals

Age-matched (~ 4 months old) male and female ATM deficient mice were used as previously described [10]. Heterozygous knockout (hKO) and wild type (WT) ATM mice, purchased from the Jackson Laboratory, were of 129xblack Swiss hybrid background. Genotyping was performed by polymerase chain reaction (PCR) using primers suggested by the Jackson Laboratory. The absence of both ATM alleles produces a lethal phenotype at ~2 months of age mainly due to thymic lymphomas [12,13]. 

### Ethics statement

The investigation conforms to the *Guide for the Care and Use of Laboratory Animals* published by the US National Institutes of Health (NIH Publication No. 85-23, revised 1996). All of the experiments were performed in accordance with the protocols approved by the East Tennessee State University Animal Care and Use Committee. 

### Myocardial infarction

MI and measurements were performed as previously described [14–16]. The left anterior descending coronary artery was occluded using a 7-0 mm silk suture. Sham animals underwent the same surgery without ligation of the coronary artery. 

### Echocardiography

Transthoracic two-dimensional M-mode echocardiography was performed as previously described [10,11]. All echocardiographic assessments and measurements were performed by the same investigator. A second person also performed measurements on a separate occasion using the same recordings with no significant differences in interobserver variability. 

### Morphometric analyses

Following MI, hearts were removed and arrested in diastole using KCl (30 mmol/L) followed by perfusion fixation with 10% buffered formalin. Infarct size was measured using Masson’s trichrome stained sections as previously described [14,16]. Infarct size was calculated as the percentage of LV circumference occupied by infarct scar. Infarct thickness was calculated using mid-myocardial slides, averaging three equally spaced measurements along the infarct wall. Cross sections (4µm thick) were stained with Masson’s trichrome for the measurement of fibrosis using Bioquant image analysis software (Nashville,TN).

### Apoptosis

To detect apoptosis, TUNEL-staining was carried out as previously described [10,11]. Hoechst 33258 (10 μM; Sigma) staining was used to count the total number of nuclei. Apoptosis was calculated as the percentage of apoptotic cardiac cell nuclei / total number of nuclei. To identify apoptosis associated with cardiac myocytes, the sections were immunostained using α-sarcomeric actin antibodies (1:50, 5C5 clone; Sigma, St. Louis, MO). TUNEL-positive nuclei that were clearly seen within cardiac myocytes were counted. The number of apoptotic myocyte nuclei was counted, and index of apoptosis was calculated as the percentage of apoptotic myocyte nuclei/total number of nuclei. In isolated cells, the percentage of TUNEL-positive cells (relative to total myocytes) was determined by counting ~200 cells in 10 randomly chosen fields per coverslip for each experiment.

### Immunohistochemistry

Sections (4µm thick) were deparrafinized and stained with anti-α-smooth muscle actin (α-SMA) as described [11]. The sections were visualized using fluorescent microscopy (Nikon) and images were acquired using Retiga 1300 color-cooled camera. Images were quantitatively analyzed using Bioquant Image analysis software (Nashville, TN). 

### Western blot analysis

LV lysates were prepared in RIPA buffer as previously described [17]. Protein lysates (50 μg) were separated by SDS-PAGE (10%) and transferred to a PVDF membrane (240 mA, 2.5 h). The membranes were incubated with antibodies against p-p53 (serine-15; Cell Signaling), MMP-9 and MMP-2 (Millipore), TIMP-2 and TIMP-4 (Chemicon), and SOD-2 (Santa Cruz). Membranes were stripped and probed with GAPDH (Santa Cruz) as a protein loading control. Band intensities were quantified using Kodak photodocumentation system (Eastman Kodak Co.). The data are presented as fold change vs WT-sham. 

### In-gel zymography

In gel zymography was performed on 50 μg of LV lysates from WT-MI and hKO-MI hearts as previously described [18]. Clear and digested regions representing MMP-2 and MMP-9 activity were quantified using a Kodak documentation system. 

### Cell isolation, culture and treatment

Adult rat ventricular myocytes (ARVMs) were isolated as previously described [19]. ARVMs were plated in Dulbecco’s modified Eagle’s medium (DMEM; Mediatech) supplemented with HEPES (25 mM), BSA (0.2%), creatine (5 mM), L-carnitine (2 mM), taurine (5 mM) and 0.1% penicillin-streptomycin at a density of 30–50 cells/mm^2^ on coverslips precoated with laminin (1μg/cm^2^). ARVMs cultured for 24 h were treated with KU-55933 (KU), a specific inhibitor of ATM [20], for 24 h. Apoptosis was measured using TUNEL-assay as described above. 

### Detection of oxidative stress

To detect oxidative stress, ARVMs were treated with KU (0.1 µM and 1 µM) for 3h. Cells were then stained using total reactive oxygen species (ROS)/superoxide detection kit (Enzo Life Sciences) and visualized using fluorescent microscopy. The number of ROS-positive cells (relative to total myocytes) was determined by counting ~100 cells in 10 randomly chosen fields per coverslip for each experiment.

### Statistical analyses

Data are represented as mean ± SEM. Data were analyzed using student’s t test or one-way analysis of variance (ANOVA) and a post hoc Tukey’s test. Probability (p) values of <0.05 were considered to be significant.

## Results

### Morphometric studies and mortality

**Figure 1:**
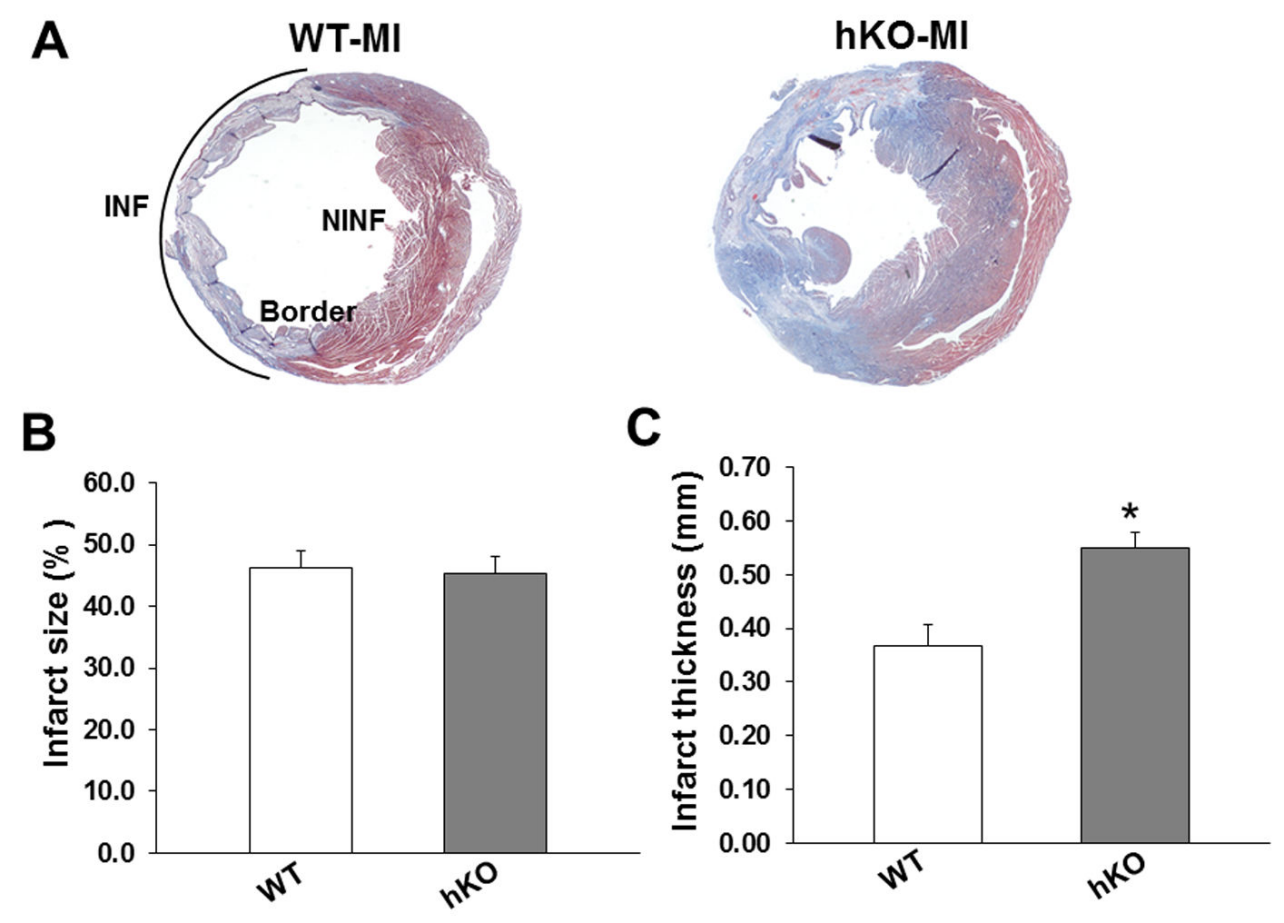
Infarct size and thickness Masson’s trichrome stained sections from WT and hKO hearts were analyzed for the measurement of infarct size and thickness. A. Transverse sections of the mid-myocardium from WT and hKO hearts post-MI. Quantitative analysis of infarct size (B) and thickness (C) as measured from trichrome stained hearts; *p<0.05 vs WT; n=5-7.

Body weights remained unchanged among the sham and MI groups. Heart weight (HW) and HW to body weight ratios were increased in both MI groups (p<0.001 vs sham; n=6-12; Table 1) with no significant difference between the two MI groups. The mortality rates 7 days post-MI were 35% and 18% in WT and hKO mice, respectively. Masson’s trichrome staining of the mid-LV sections is shown in Figure 1A. Infarct size measured as a percentage of the LV circumference occupied by scar tissue was not different between the WT-MI and hKO-MI groups (p=NS, Figure 1B). However, infarct thickness measured from mid-myocardial sections was significantly greater in hKO-MI group versus WT-MI (Figure 1C). 

**Table 1:** Morphometric Measurements.

	WT-Sham (n=6)	hKO-Sham (n=7)	WT-MI (n=10)	hKO-MI (n=12)	p
BW	24.41 ± 0.96	26.49 ± 1.71	23.89 ± 0.76	23.43 ± 0.61	
HW	125.22 ± 7.74	139.08 ± 12.40	153.93 ± 5.27*	158.50 ± 5.93*	<0.001
HW/BW	5.11 ± 0.12	5.25 ± 0.04	6.48 ± 0.33*	6.86 ± 0.43*	<0.001

Values are mean ± SEM; *comparison between sham and MI group.

### Echocardiographic studies

**Figure 2:**
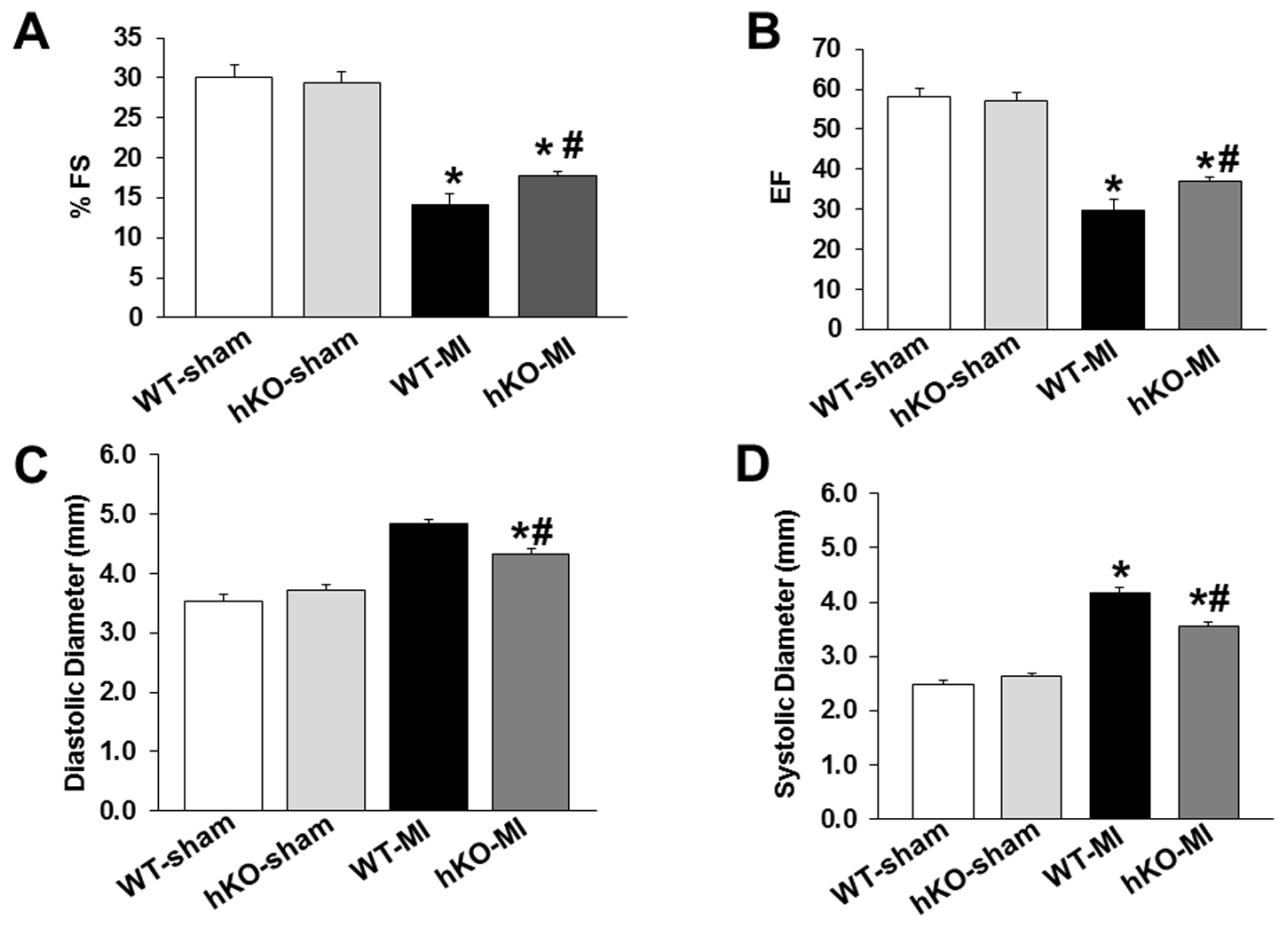
ATM deficiency improves LV function 7 days post-MI. MI was performed in WT (n=10) and ATM hKO (n=12) mice. Indices of cardiac function (percent fractional shortening, %FS; ejection fraction, EF) and structure (LV end diastolic diameter, LVEDD; LV end systolic diameter, LVESD) were measured using echocardiography 7 days after MI. A. %FS; B. EF; C, LVEDD; D. LVESD; *p<0.05 vs sham; ^#^p<0.05 vs WT-MI; n=10-12.

No significant differences in the echocardiographic parameters were observed between the two sham groups. M-mode echocardiography revealed a significant decrease in percent fractional shortening (%FS) and ejection fraction (EF) in both MI groups when compared to their respective sham groups. However, the decrease in %FS and EF was significantly greater in the WT-MI group when compared to hKO-MI (^#^p<0.05 vs WT-MI; Figure 2A & B). MI increased LV end systolic (LVESD) and diastolic (LVEDD) diameters in both MI groups. However, the increase in LVEDD and LVESD was significantly lower in hKO-MI when compared to the WT-MI group (^#^p<0.05 vs WT-MI; Figure 2C & D). 

### Fibrosis and Apoptosis

**Figure 3:**
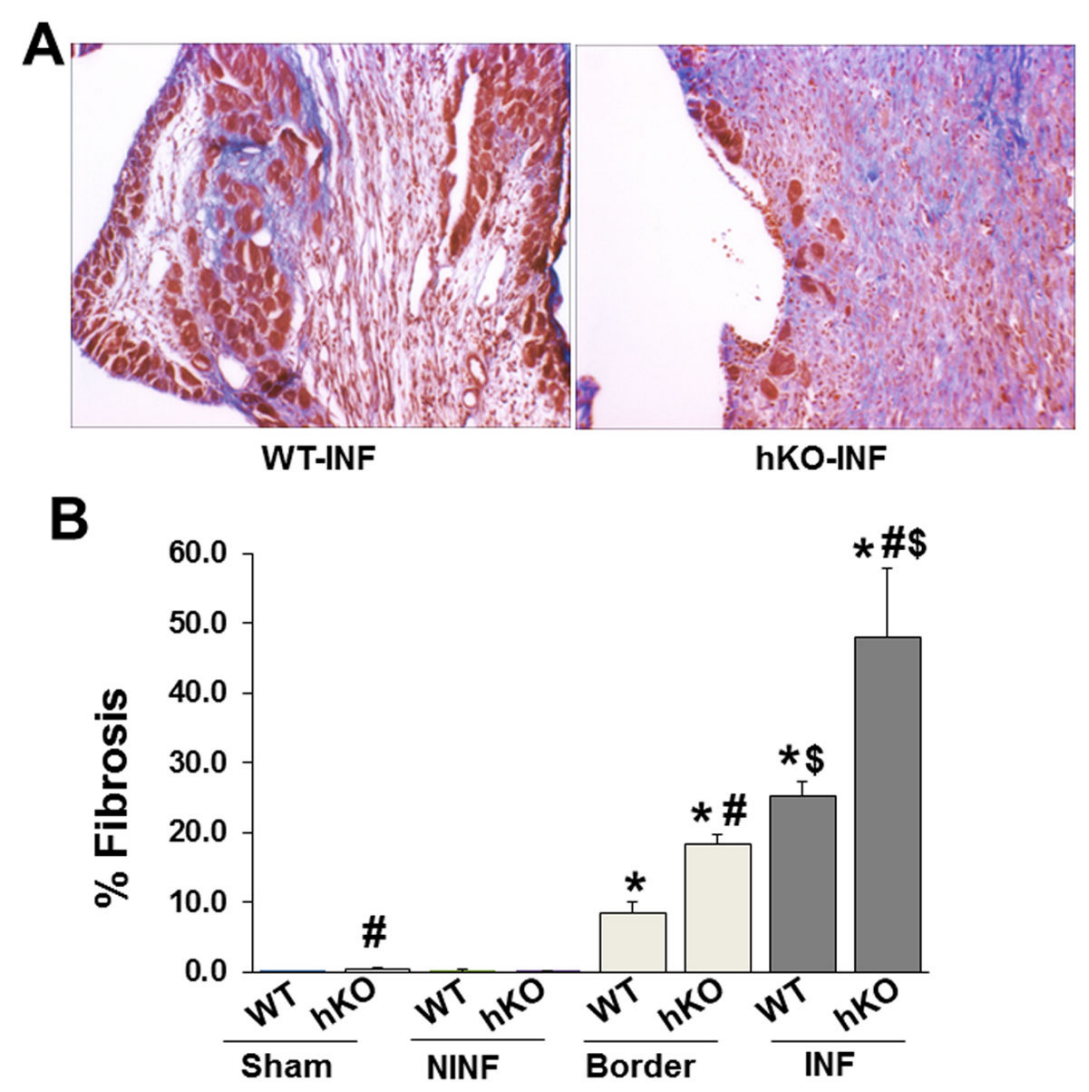
Analysis of fibrosis. Masson’s trichrome stained sections of the heart were used for quantitative measurement of fibrosis. A. Masson’s trichrome-stained sections demonstrating fibrosis in WT and hKO mice 7 days post-MI. B. Quantitative analysis of fibrosis. NINF, non-infarct LV region; INF, infarct; *p<0.05 vs sham; #p<0.05 comparisons between WT and hKO groups; ^$^p<0.05 vs border; n=6-7.

Quantitative analysis of fibrosis using trichrome stained sections revealed increased fibrosis in hKO-sham group vs WT-Sham. MI increased fibrosis in the border and infarct LV regions of both groups when compared to their respective non-infarct LV regions. Interestingly, the level of fibrosis was greater in the border and infarct regions of hKO-MI group when compared to the WT-MI (Figure 3 A&B). 

**Figure 4:**
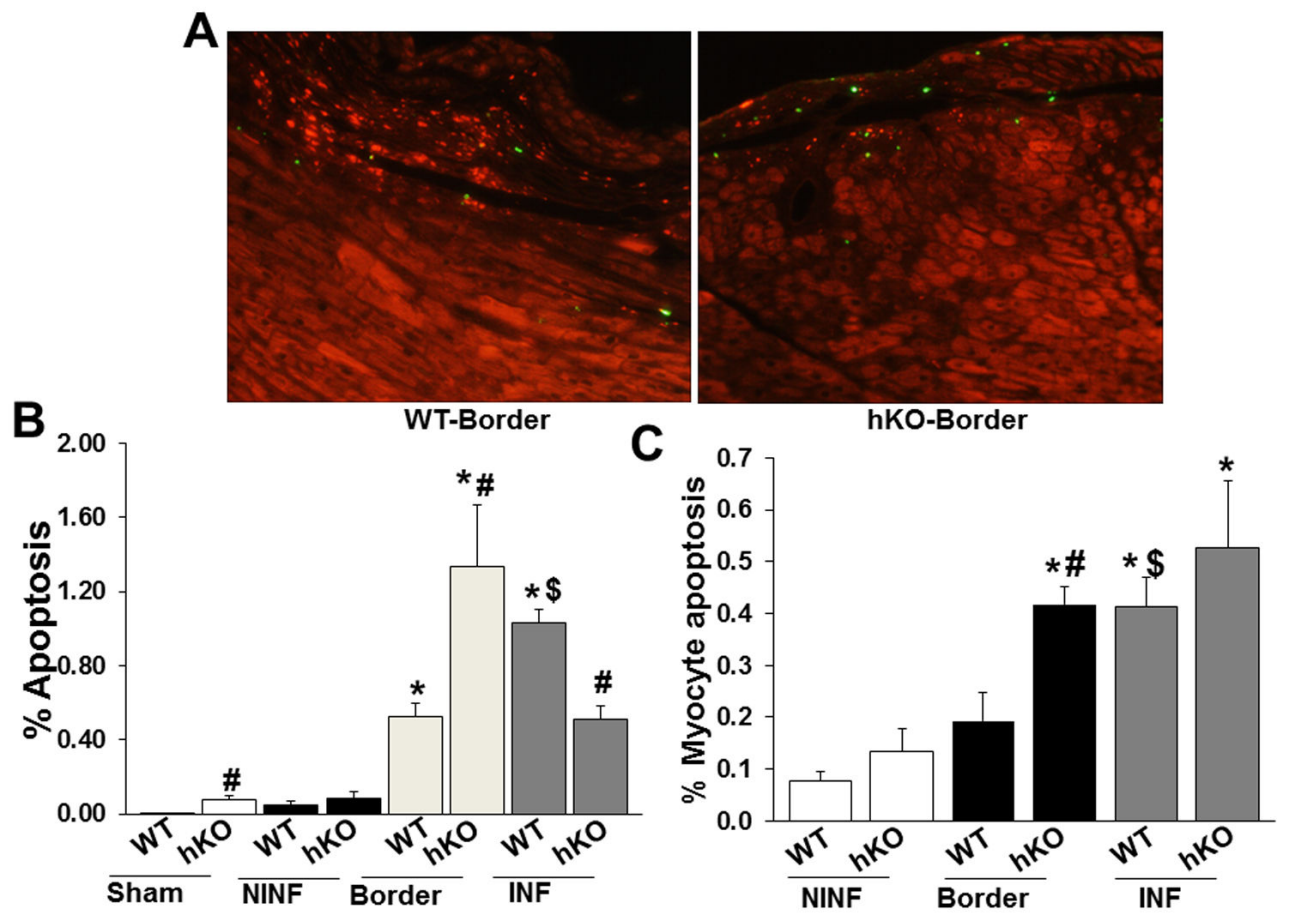
Analysis of apoptosis. A. TUNEL-stained images from the border regions of WT and hKO hearts post-MI. B. Quantitative analysis of cardiac cell apoptosis in the non-infarcted (NINF), border and infarct (INF) regions of WT and hKO mice 7 days post-MI. C. Quantitative analysis of myocyte apoptosis in the NINF, border and INF regions of WT and hKO mice 7 days post-MI. *p<0.05 vs sham; #p<0.05 comparisons between WT and hKO groups; ^$^p<0.05 vs border; n=4.

Analysis of apoptosis using TUNEL-staining assay revealed increased apoptosis in the hKO-sham vs the WT-sham group. The number of apoptotic cells and myocytes remained unchanged in the non-infarct LV regions when compared to the sham groups (Figure 4 B&C). MI increased the number of apoptotic cells in the border and infarct LV regions of both groups when compared to the sham and non-infarct LV regions (Figure 4 A&B). In the border area, the number of apoptotic cells as well as myocytes was significantly higher in hKO vs WT group. In the infarct LV region, the number of apoptotic cells was significantly lower in hKO vs WT group (Figure 4B), while the number apoptotic myocytes remained unchanged between WT and hKO (Figure 4C). 

### Expression of α-smooth muscle actin (α-SMA)

**Figure 5:**
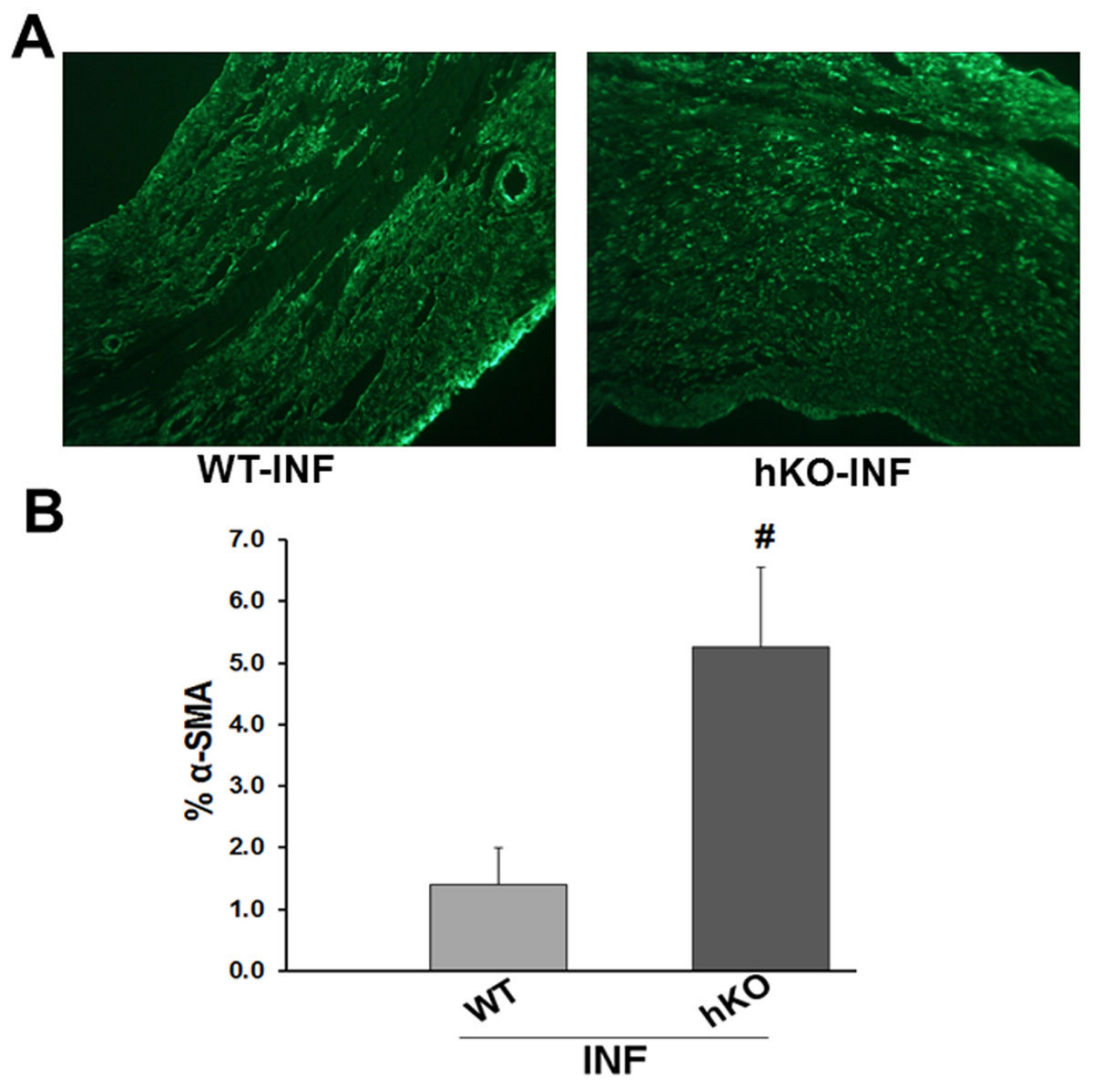
Expression of α-smooth muscle actin (α-SMA). A. α-SMA-stained images from the infarct LV regions of WT and hKO hearts post-MI. B. Quantitative immunohistological analysis of α-SMA expression in the infarct (INF) region of WT and hKO mice 7 days post-MI. #p<0.05 vs WT-INF; n=4.

Expression of α-SMA serves as a marker for the differentiation of fibroblasts into myofibroblasts [21–23]. MI increased α-SMA expression in the infarct LV regions of both groups (Figure 5A). Quantitative immunohistochemical analysis of heart sections revealed increased α-SMA expression in the infarct LV region of hKO when compared to the WT group (Figure 5B). 

### Expression of matrix metalloproteinases (MMPs) and tissue inhibitors of MMPs (TIMPs)

**Figure 6:**
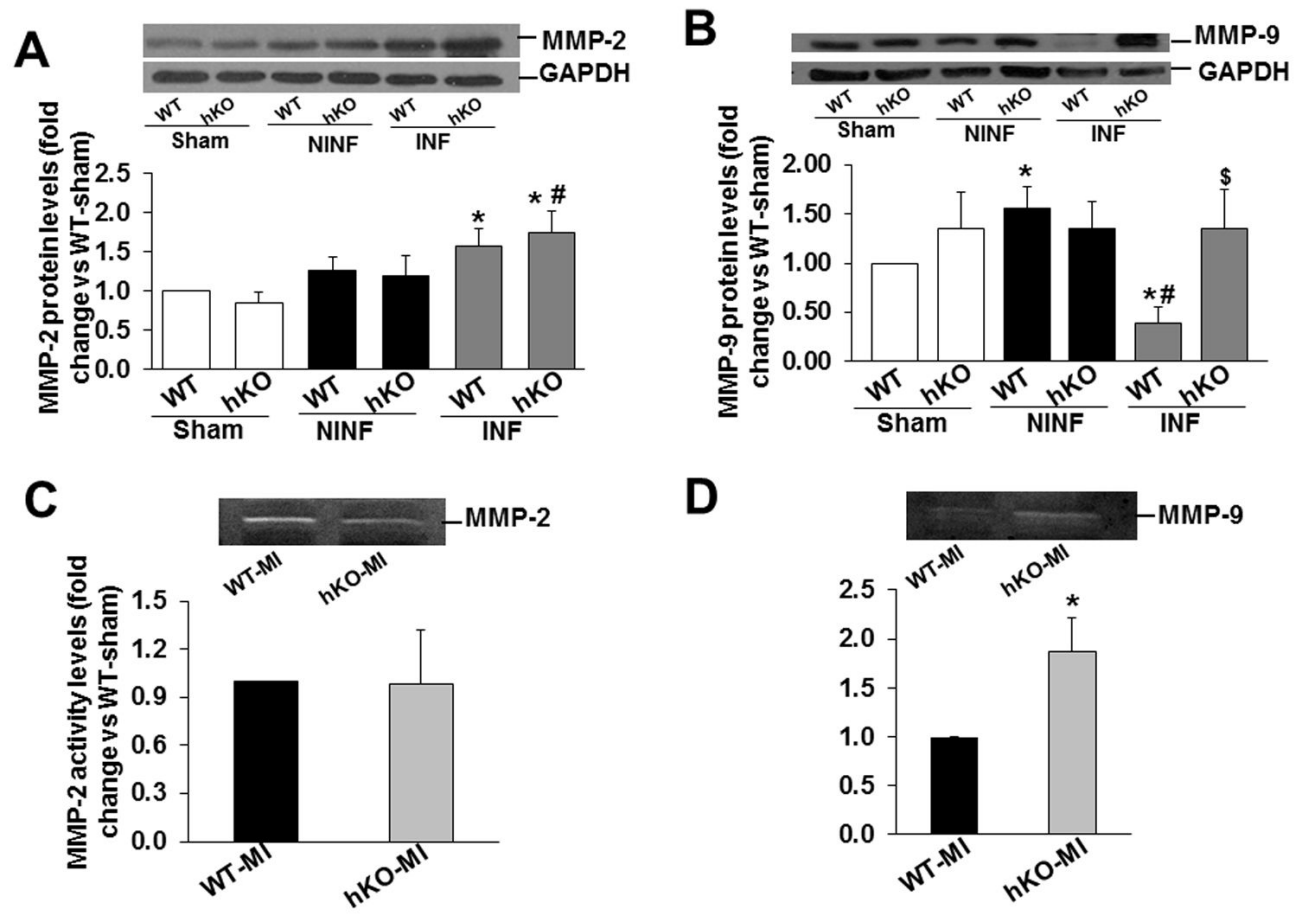
Expression and activity of MMPs. A & B. Total LV lysates (50 µg), prepared from sham and non-infarct (NINF) and infarct (INF) LV regions, were analyzed by western blot using anti-MMP-2 (A) and anti-MMP-9 (B) antibodies. The upper panels show autoradiograms indicating immunostaining for MMP-2, MMP-9, and GAPDH. The lower panels exhibit quantitative analyses of MMP-2, MMP-9 normalized to GAPDH. *p<0.05 vs sham; ^#^p<0.05 vs NINF; ^$^p<0.05 vs WT-INF; n=7. C&D. Total LV lysates (50 µg), prepared from the infarct LV regions were analyzed by in-gel zymography. C. MMP-2 activity. D. MMP-9 activity. *p<0.05 vs WT-MI; n=3.

Western blot analyses of LV lysates revealed no significant increase in MMP-2 protein levels in the non-infarct LV regions of both MI groups when compared to sham. MMP-2 protein levels were significantly higher in the infarct region of both MI groups when compared to sham. In the hKO group, the increase in MMP-2 protein levels was significantly greater in the infarct region when compared to the non-infarct region (Figure 6A). MMP-9 protein levels were increased in the non-infarct region and decreased in the infarct region of the WT group when compared to sham. No such changes in MMP-9 protein levels were observed in the hKO group. In the infarct region, MMP-9 protein levels were significantly greater in the hKO group when compared to the WT (Figure 6B). Analysis of MMPs activity using in-gel zymography showed no difference in MMP-2 activity the infarct LV regions between the two MI groups (Figure 6C). However, MMP-9 activity in the hKO was significantly higher in the hKO group when compared to WT (Figure 6D).

**Figure 7:**
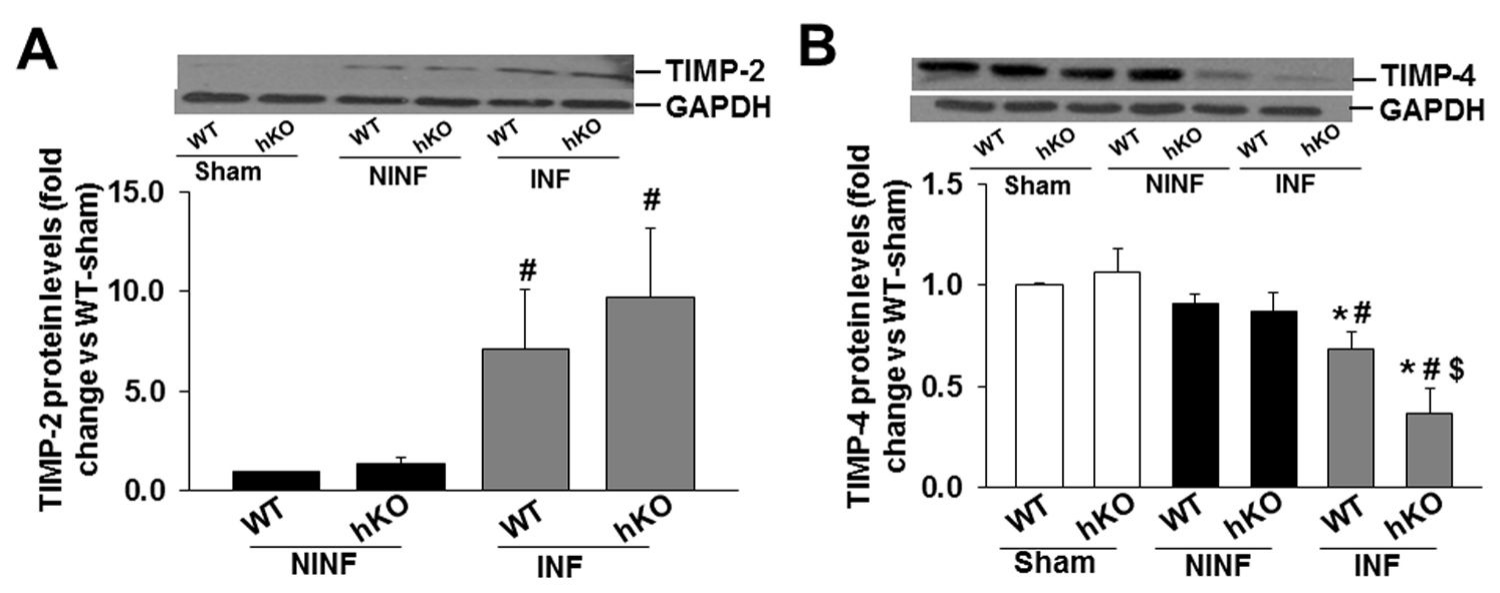
Expression of TIMPs. Total LV lysates (50 µg), prepared from sham and non-infarct (NINF) and infarct (INF) LV regions, were analyzed by western blot using anti-TIMP-2 (A) and anti-TIMP-4 (B) antibodies. The upper panels show autoradiograms indicating immunostaining for TIMP-2, TIMP-4, and GAPDH. The lower panels exhibit quantitative analyses of TIMP-2, TIMP-4 normalized to GAPDH. *p<0.05 vs sham; ^#^p<0.05 vs NINF; ^$^p<0.05 vs WT-INF; n=7.

TIMP-2 is suggested to inhibit MMP-2 activity [4], while TIMP-4 is predominantly expressed in the heart [24] . Western blot analyses showed no immunostaining for TIMP-2 in the sham groups. MI increased TIMP-2 protein levels in the non-infarct and infarct regions of the heart in both groups. However, TIMP-2 protein levels were significantly greater in the infarct region when compared to the non-infarct LV region with no significant difference between the WT and hKO groups (Figure 7A). On the contrary, TIMP-4 protein levels were clearly present in both the sham groups. MI decreased TIMP-4 protein levels in the infarct LV region of both groups. TIMP-4 protein levels were significantly lower in hKO group when compared to WT (Figure 7B). 

### Expression and phosphorylation of apoptosis-related proteins

**Figure 8:**
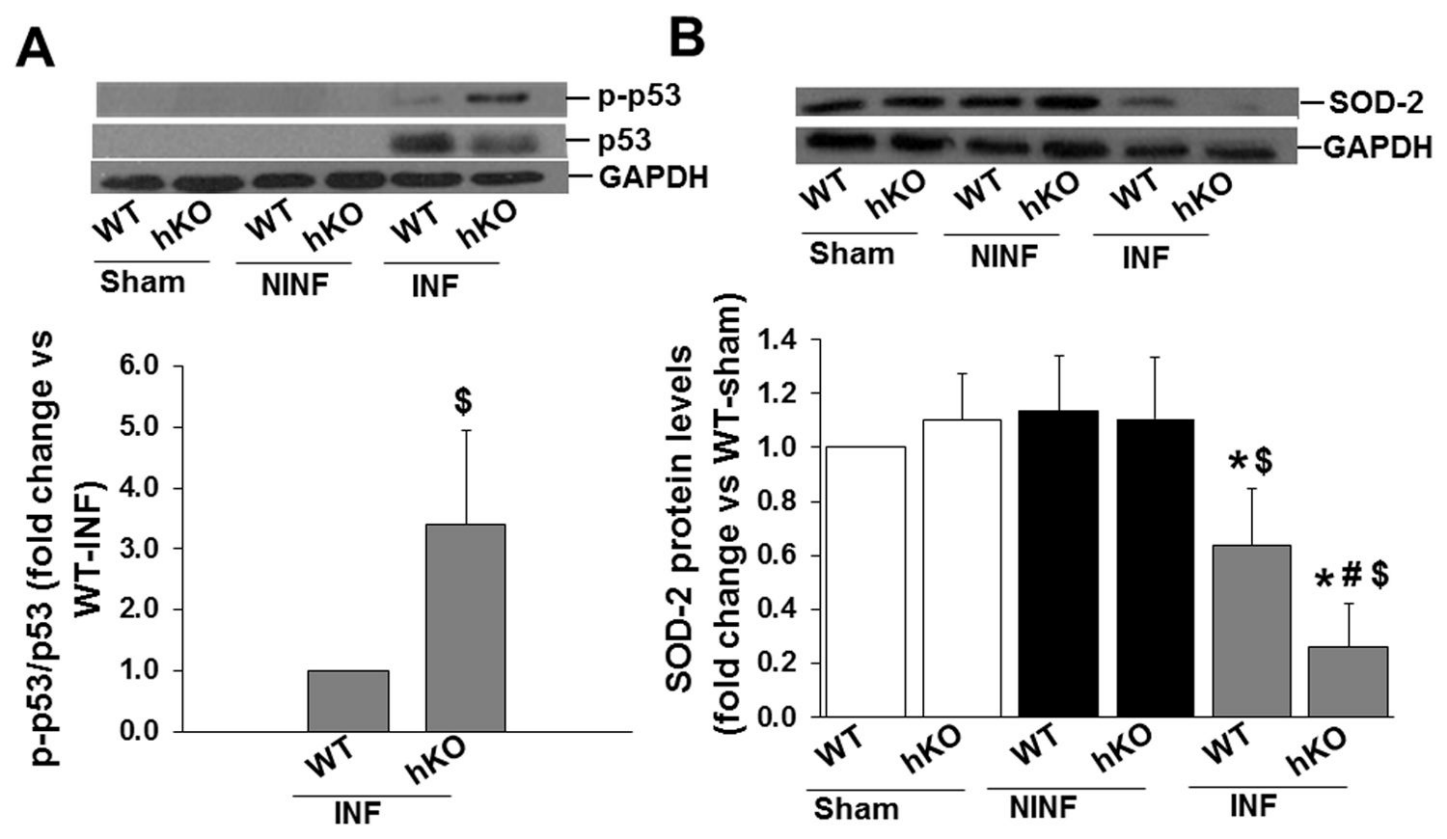
Expression and phosphorylation of apoptosis related proteins. A. Total LV lysates were analyzed by western blot using phospho-specific (serine-15) p53 or total p53 antibodies. Protein loading in each lane is indicated by GAPDH immunostaining. $p<0.05 vs WT-INF; n=7. B. Total LV lysates were analyzed by western blot using anti-SOD-2 antibodies. Protein loading in each lane is indicated by GAPDH. *p<0.05 vs sham; ^$^p<0.05 vs NINF; ^#^p<0.05 vs WT-INF; n=7.

ATM phosphorylates p53 (serine-15) following DNA damage [25]. Western blot analyses of LV lysates using anti-p53 antibodies showed no immunostaining for p53 in the sham or non-infarct LV regions of WT or hKO groups. Phosphorylation of p53 (serine-15) was only observed in the infarct regions with a greater increase in the hKO-MI group (Figure 8A). 

Deficiency of ATM is suggested to associate with increased oxidative stress [26]. Intrinsic mitochondrial abnormalities are also reported in thymocytes lacking ATM [27]. Western blot analysis of mitochondrial antioxidant protein manganese superoxide dismutase (SOD-2) demonstrated no change in SOD-2 protein levels in the sham and non-infarct LV regions of the heart in both groups. MI significantly decreased SOD-2 protein levels in the infarct LV of both groups. However, the decrease in SOD-2 protein levels was significantly higher in hKO group vs WT (Figure 8B). 

### Oxidative stress and apoptosis in ARVMs

**Figure 9:**
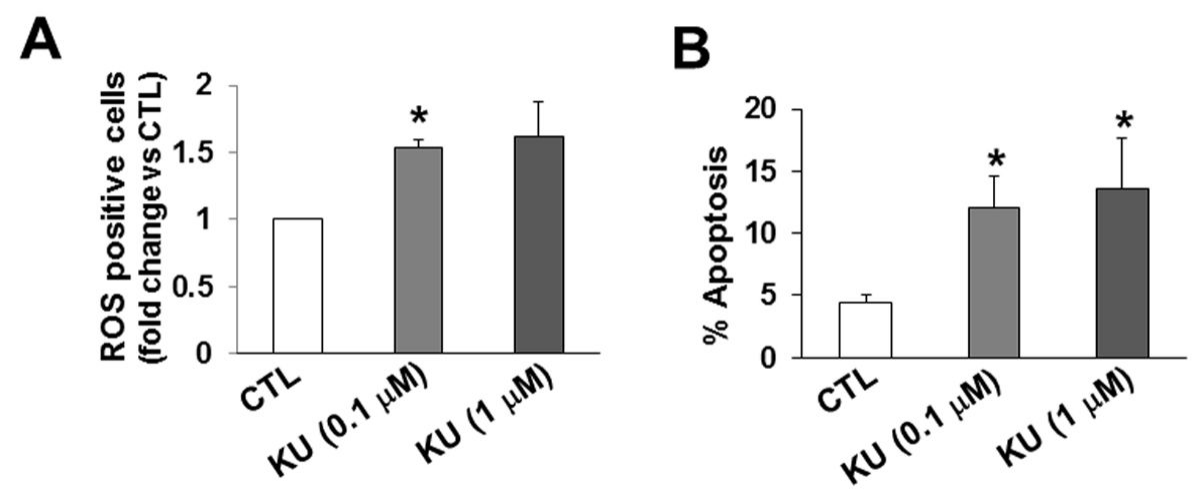
Inhibition of ATM increases the number of ROS-positive ARVMs and induces apoptosis. ARVMs were treated with KU-55933 (KU) for 3 h (A) or 24 h (B). A. Cells were stained using ROS-detection kit and ROS-positive cells were counted using fluorescent microscopy. *p<0.05 vs control (CTL); n=3. B. TUNEL-assay was used to count the number of apoptotic ARVMs. *p<0.05 vs CTL; n=3.

Inhibition of ATM using KU-55933 (KU) is shown to increase reactive oxygen species (ROS) in cancer cells [28]. To investigate if inhibition of ATM also increases ROS in myocytes, ARVMs were treated with KU (0.1 µM and 1 µM) for 3 h. Analysis of ROS-positive ARVMs using fluorescent microscopy showed increased number of ROS-positive ARVMs at both concentrations of KU (Figure 9A). To investigate if inhibition of ATM induces apoptosis, ARVMs were treated with KU (0.1 µM and 1 µM) for 24 h. Measurement of apoptosis using TUNEL-assay showed that KU at 0.1 µM and 1 µM concentrations significantly increases the number of apoptotic ARVMs (Figure 9B). 

## Discussion

Previous studies from our lab have shown that lack of ATM induces structural and functional changes in the heart [10,11]. A major finding of this study is that deficiency of ATM attenuates LV dysfunction and dilatation 7 days post-MI. Although infarct size were comparable between the WT and hKO mice, infarct thickness was greater in the hKO mice. Deficiency of ATM associated with increased expression of α-SMA and fibrosis. Cardiac cell apoptosis was lower in the infarct LV region of ATM deficient mice. However, apoptosis was significantly higher in the border area of the infarct in ATM deficient mice. ATM deficiency also associated with changes in the expression of fibrosis- and apoptosis-related proteins. In vitro, inhibition of ATM increased the number of ROS-positive ARVMs and induced apoptosis. The results presented here suggest that ATM plays a multifaceted role in remodeling pathways following myocardial infarction. 

Systolic dysfunction is characterized by decreased cardiac contractility and pumping capacity of the heart. In general, MI results in severe LV dilation and systolic dysfunction [29]. The data presented here demonstrate that ATM deficient mice suffer to a lesser degree from impaired systolic function following MI. The study also provides evidence that deficiency of ATM affects the infarct scar. We observed that the infarct size remained unchanged between WT and ATM deficient mice. However, infarct thickness was greater in the ATM deficient mice. Fibrosis and expression of α-SMA, a marker of myofibroblasts, was significantly higher in the ATM deficient heart after MI. Our findings suggest that myofibroblasts, a major cell type involved in the deposition of fibrosis, may escape the apoptotic death during the granulation phase and contribute to the increase in infarct wall thickness in the ATM deficient mice. This may in turn decrease LV dilation and dysfunction. Of note, cardiac cell apoptosis in the infarct LV region of ATM deficient mice was lower when compared to that in the WT. Increased infarct thickness with reduced apoptosis in granulation tissue cells is previously described in Angiotensin II type 1A receptor KO (AT1AKO) 7 days post-MI [30]. Expression of soluble transforming growth factor-β (TGF-β) type II receptor, a competitive inhibitor of TGF-β, led to a greater infarct thickness and smaller LV circumference [31]. Therefore, it is conceivable that ATM signaling may involve Angiotensin II and/or TGF-β axis for its effect on infarct thickness, LV circumference and apoptosis. Further investigations are warranted to clarify the role of these molecules in ATM signaling.

Cardiac structure consists of various cell types whose function is to promote contractility of the heart. Following injury, macrophages and other immune cells initiate a healing process. Once the damaged cells have been removed, activation of myofibroblasts helps promote scar tissue formation. This response is suggested to be associated with increases in α-SMA [21,32]. We observed greater increase in α-SMA expression in the infarct region of ATM deficient mice. Increased α-SMA may help explain the presence of increased fibrosis in the infarct region of ATM deficient mice. 

Cardiac cell apoptosis increases in the infarct and border areas and to a smaller extent in the non-infarcted areas of the heart after MI [6,33]. Early activation of apoptosis is a necessary step in remodeling as it allows room for entry of immune repair cells following injury [1,34,35]. The infarct region is a predictor of post-MI prognosis as it can relate to infarct expansion. ATM deficient mice exhibited a greater increase in apoptosis in the border region. This may lead to a greater infarct expansion and worse prognosis late post-MI [1,3,36]. Likewise, increased granulation tissue cell apoptosis in WT-MI hearts may reflect timely removal of unhealthy cells, thereby enhancing the repair process. 

The matrix metalloproteinases (MMPs) are endopeptidases that are present within the myocardium. Changes in MMP abundance is shown to be associated with changes in extracellular matrix deposition (ECM) and LV remodeling post-MI, including increased LV dilation and cardiac rupture [37–41]. The ECM is a critical component in the restructuring of the heart after MI. Using promoter reporter constructs, Mukherjee et. al. showed that MMP-2 promoter activation peaks in the MI region 7 days post-MI, while MMP-9 promoter activation was highest in the border region at 7 and 14 days post-MI [42]. Consistent with these findings, we observed increased MMP-2 protein levels in the MI regions of both groups when compared to their respective sham groups. No difference in MMP-2 protein levels and activity in the infarct LV region suggest that increased fibrosis in hKO mice occurs via MMP-2-independent mechanism. MMP-9 protein levels were significantly higher in the non-infarct LV region of WT group when compared to sham. However, we observed decreased MMP-9 protein levels in the infarct LV region of WT group. No such changes in MMP-9 protein levels were observed in the hKO group. MMP-9 activity was higher in the infarct LV region of hKO when compared to WT. Higher MMP-9 protein levels and activity suggest involvement of MMP-9 in ECM deposition and LV remodeling during ATM deficiency. The decreased MMP-9 protein levels observed in this study in the WT infarct region may reflect localization and/or timing of the remodeling events [43].. Tao et al. has shown that MMP-9 activity increases as early as 1 day post-MI and reaches a maximum by 2 days, then gradually decreases. MMP-2 activity starts to increase 4 days post-MI, reaching a maximum by 7 days [41]. 

TIMP’s are traditionally believed to function solely as inhibitors of active MMPs [4]. TIMP-2 is suggested to have maximum affinity for MMP-2 [44]. In the heart, MMP-9 and TIMP-4 are suggested to play a key in the myocardial remodeling. In cardiac myocytes, the effects of MMP-9 on voltage-induced contraction can be reversed by TIMP-4 [45]. We observed increased TIMP-2 expression in the infarct region of both groups to a similar extent. However, TIMP-4 protein levels were significantly lower in the ATM deficient mice. ATM deficient mice also exhibited increased MMP-9 protein levels and activity in the infarct region when compared to their WT counterparts. Increased MMP-9 activity is expected to correlate with decreased fibrosis in ATM deficient mice. However, we observed increased fibrosis in the infarct and remote regions of ATM deficient mice. Recent evidence suggests additional roles for TIMPs independent of their function as MMP inhibitors [36]. Therefore, it is plausible that changes in TIMP protein levels observed in the infarct region of WT and ATM deficient mice could function in the remodeling processes of the heart independent of MMP-2 and -9. 

ATM deficiency results in impaired repair of double-stranded DNA breaks and increased oxidative stress [26,46]. Deceased SOD levels impair the cell’s response to handle reactive oxygen species following myocardial injury [47,48]. ATM is also known to phosphorylate p53 on serine-15 resulting in its stabilization. Stabilization of p53 increases its transcriptional activity, leading to increased apoptosis [49]. Here we observed greater increase in phosphorylation of p53 in the infarct region of ATM deficient hearts. On the other hand, SOD-2 protein levels were lower in ATM deficient hearts, suggesting enhanced oxidative stress during ATM deficiency. Enhanced oxidative stress and apoptosis was also observed in ARVMs during inhibition of ATM using KU-55933. It can be argued that increased p53 phosphorylation and oxidative stress should increase apoptosis in the infarct region of ATM deficient mice. We did observe increased apoptosis in the border area of ATM deficient mice. However, apoptosis was lower in the infarct region of ATM deficient mice. Inhibition of ATM and ATR failed to prevent H_2_O_2_-induced phosphorylation of p53 in neonatal cardiac myocytes [50]. In addition, graded increases in the level of oxidative stress induce a graded phenotype shift in cardiac myocytes, from hypertrophy at low levels of oxidative stress, to apoptosis at high levels of oxidative stress [51]. Therefore, it is conceivable that p53 phosphorylation during ATM deficiency involves signaling pathways independent of ATM, and increased oxidative stress during ATM deficiency promotes cell growth in the infarct LV region at this time point. 

### Conclusion and study limitations

The data presented here provide evidence that ATM has the potential to modulate infarct tissue dynamics. It alters the infarct structure by affecting apoptosis, fibrosis, and expression of α-SMA. It should be emphasized that our data on investigating the role of ATM in myocardial remodeling post-MI are obtained 7 days post-MI. Changes in the size and thickness of infarct scar can eventually affect infarct expansion and contribute to the diastolic dysfunction. Therefore, it is possible that increased fibrosis (stiffness) and apoptosis (in the border area) in ATM deficient mice may associate with earlier diastolic dysfunction if the study time points are extended beyond 7 days post-MI. 
